# Editorial: Advancing cancer imaging technologies: bridging the gap from research to clinical practice

**DOI:** 10.3389/fonc.2026.1775769

**Published:** 2026-04-10

**Authors:** Richa Vaish, Abhishek Mahajan

**Affiliations:** 1 Head and Neck Surgical Oncology, Tata Memorial Hospital, Homi Bhabha National Institute, Mumbai, India; 2 Radiology, The Clatterbridge Cancer Centre NHS Foundation Trust, Liverpool, United Kingdom

**Keywords:** AI, artificial intelligence, functional image analysis, oncology, radiogenomic imaging, radiomics, technological innovation

## Introduction

Imaging plays a crucial role in the management of cancer, from screening and diagnosis through staging, treatment planning, and follow-up to assess response. The imaging landscape is rapidly evolving, with functional imaging and deep learning now being incorporated into clinical practice. With advances in imaging, it is equally essential that these be appropriately applied in clinical practice. In the era of precision medicine, these developments have the potential to revolutionize treatment. Accurate diagnosis, characterization, and monitoring of response are core tenets of cancer management. Imaging relies on visual assessment, which can be enhanced substantially by computational analysis and multilayered data. Artificial intelligence can interpret qualitative data in detail to predict tumour genotype and biological course from radiographic phenotype and clinical outcome (1, 2). It can identify patterns which the human eye may not capture. Based on these patterns, it not only detects lesions early but also provides insight into the subtype, aggressiveness, mutational status and stage (3–5). However, it is limited by the absence of a standardized protocol and clear guidelines. It is crucial to develop an algorithm using balanced, well-labeled data, and to validate it for reproducibility and generalizability to improve clinical applicability (5). Additionally, ethical considerations must be clearly defined to foster collaboration and research (6).

Functional imaging has garnered interest due to its non-invasive, quantitative nature for studying tumour physiology, molecular processes, tumour molecules, and metabolites. It can complement structural imaging to improve detection and diagnosis and to assess response. It surpasses traditional imaging by deciphering tumour gene expression and tracking cells and therapeutic drugs (7). Functional imaging has also gained importance as it can visualize, characterize, and quantify biological processes at the cellular, subcellular, and molecular levels. Molecular functional imaging helps visualize and quantify biochemical and physiological processes during tumorigenesis. Radiogeomics, the integration of radiomics with genomics, further helps elucidate tumour genetics (8, 9). This is particularly relevant in the era of targeted therapy, where the molecular imaging-based probes can target cell signaling pathways (10). It is a crucial tool for studying newer receptor-targeted and gene therapies, as well as for cancer stem cell research, which requires the development of biotechnology, information technology, and basic translational science to achieve optimal outcomes (11).

Radiomics can extract high-dimensional quantitative features from routine imaging, which helps in diagnosis and risk stratification (12). It also supports molecular characterization and outcome prediction. It has been explored across various tumour subsites, including pediatric brain tumours, to identify tumour subtypes, grading, molecular subgroups, and mutations (13, 14). Deep-learning-based radiogenomic (DLR) models and radiomic signatures have been used to identify the EGFR mutation status of non-small cell lung cancer from CT imaging (15). These developments in imaging have the potential to boost clinical decision-making in the era of precision medicine.

Despite these strides in development in artificial intelligence and radiomics, there is a need to overcome translational barriers to bridge the gap from research to clinical implementation. There are several practical barriers, such as sustainability and system maintenance requiring updates and recalibrations, a lack of standardization and external validation, particularly in algorithms developed on a single institutional retrospective dataset, and regulatory challenges with authorities still defining the approval process for adaptive algorithms (12, 16, 17). The issue is further compounded by a lack of robust data on cost-effectiveness and the need for high initial investments in digital infrastructure, personnel training, and information technology setup (16).

In addition, there are ethical concerns about data privacy as AI use expands, necessitating advances in data encryption. The models and algorithms are developed on the training dataset. The training dataset may not cover the entire spectrum of the patient population due to underrepresentation of racial, ethnic, or socioeconomic groups, and hence may not be suitable for this cohort of patients. There may be reluctance among clinicians to use AI in clinical practice, as it operates like a black box: it does not provide a rationale for its predictions (16, 17). There is a need to overcome these limitations to translate the development into the day-to-day clinical practice. The Research Topic presents a rich collection of imaging articles that cover conventional and advanced imaging techniques and the evolving role of deep learning in cancer radiology. The Research Topic highlights the multitude of imaging protocols with the potential to impact the clinical practice of cancer management. The section below provides brief glimpses of these articles.

## Articles on the Research Topic

Dynamic changes in brain metastasis are critical to treatment effectiveness. Pathology biopsy has limitations in assessing this in real time; hence, imaging plays a crucial role in guiding treatment. Li et al analyzed 114 patients with lung cancer brain metastasis (LCBM) prospectively who underwent evaluation with DCE-MRI and DWI on a 3.0T MRI scanner and looked at the parameters such as volume transfer constant (Ktrans), rate constant (Kep), extravascular extracellular volume (Ve), and plasma volume fraction (Vp), which were derived using the Extended Tofts model. The authors validated multimodal imaging for LCBM heterogeneity characterization to aid microenvironment-targeted therapy.

Tumour angiogenesis and cell proliferation directly affect tumour invasiveness and growth. Contrast-enhanced US is frequently used to assess the tumour microvessels. With the increasing incidence of non-alcoholic fatty liver disease (NAFLD), it is crucial to understand how it affects the diagnostic performance of the CEUS presentation and perfusion of focal hepatic lesions. Liu et al established VX2 tumour models in 24 rabbits with normal livers and NAFLD. The authors investigated whether NAFLD affected the microcirculation characteristics of VX2 hepatomas. The study also examines the impact of fatty liver background on tumour angiogenesis and proliferative activity.

Yuan et al studied retrospectively clinical factors and body composition metrics extracted from quantitative CT (QCT)in 127 AML patients to see if the QCT metrics impacted survival. The authors developed a nomogram based on QCT metrics and clinical-physical factors to predict prognosis in AML. Areas under the ROC curve (AUCs) for the nomogram based on lower skeletal muscle index and independent clinical factors for predicting 1- and 2-year OS were calculated.

The Research Topic also has an interesting case report on functional MR imaging of alveolar soft part sarcoma (ASPS). Li et al present a detailed analysis of multi-parameter quantitative functional MRI (fMRI) characteristics, including high cellular density, increased vascular permeability, and abundant neovascularisation, in ASPS. The fMRI also identifies local microcirculation characteristics, which provide insight into the tumour biology and prognosis.

Deep learning radiomics (DLR) has been explored for lesion identification and cancer diagnosis. Many DLR studies have analyzed intratumoral imaging for diagnosis and outcome prediction in breast cancer. Emerging evidence indicates that peritumoral regions provide supplementary information. Shen et al explored the impact of key parameter selection on improving the performance of the intratumoral-peritumoral region fusion model for distinguishing benign from malignant breast tumours. The authors constructed a DLR model using contrast-enhanced US images to identify intratumoral regions of interest, then expanded it to generate peritumoral regions of varying thicknesses, and assessed its diagnostic capability in differentiating benign from malignant tumours.

Mutations in the genes KRAS and BRAF have prognostic and therapeutic implications in colorectal cancer. The guidelines recommend that the KRAS mutation status of all suspected or confirmed metastatic colorectal cancers be determined, particularly if they are being considered for treatment with EGFR inhibitors. Mutant KRAS confers innate resistance to EGFR inhibitors. In recent years, various imaging modalities have been successfully used to predict KRAS mutation status. Radiomics has the potential to extract features from these modalities in a high-throughput manner, which can be used for prognostication and management of these tumours. Ai et al systematically reviewed the literature on KRAS mutation prediction using multimodal imaging and radiomics in these patients and compared learning algorithms, feature selection strategies, and validation metrics. The authors also summarize the role of radiomics in prediction models, focusing on deep learning versus conventional radiomics.

Hepatic Perivascular epithelioid cells tumours (PEComa) are very rare and lack well-described clinical manifestations or imaging diagnostic criteria. Li et al retrospectively analyzed clinical and imaging data from 4 patients with hepatic PEComa of varying pathological grades. The complete clinical details, laboratory assessments, imaging, histopathological, and immunohistochemical parameters were reviewed. The authors concluded that, in the absence of specific clinical and laboratory features and imaging findings of the lesions with prominent arterial-phase enhancement and thickened, tortuous vessels, this should raise suspicion of hepatic PEComa.

Thermal ablation is being explored as an alternative to surgery in benign and select malignant thyroid nodules. Intraprocedural monitoring is required to ensure complete ablation and limit complications. The thyroid parenchyma has good vascular flow; however, papillary thyroid cancer (PTC) is relatively avascular, with irregular microvascular patterns. Li et al compared micro-flow imaging (MFI) with contrast-enhanced ultrasound for monitoring post-microwave ablation. The authors analyzed 83 malignant nodules in 79 PTC cases and concluded that the effectiveness and safety of the two modalities were similar; however, cost-effectiveness and patient satisfaction were higher with MFI, making it a preferred option.

Pang et al published an interesting case report of a femoral metastatic cancer in a case of poorly differentiated squamous cell lung cancer posing a diagnostic dilemma. The patient had a fracture of the femur presenting as hip pain following minor trauma. CT imaging and ultrasound were suggestive of an avulsion fracture. On further investigation due to unsettling pain, MRI showed a pathological fracture due to metastatic cancer identified based on irregularity of the lesser trochanter, abnormal bone marrow signals and associated soft tissue mass.

Colorectal cancers can have recurrence in 30-50% patients postoperatively, and imaging protocol is crucial to detect these recurrences. Fibroblast activation protein (FAP) is overexpressed in cancer-associated fibroblasts in colorectal cancer. It has been exploited as a target for radionuclide-labeled fibroblast activation protein inhibitors (FAPIs) for PET/CT in cancer detection. However, adds a significant cost to the procedure. An alternative to this is technetium-99m (99mTc)-labeled FAPI SPECT/CT, but it has slightly lower diagnostic accuracy. Therefore, Sun et al investigated the diagnostic value of dual-phase 99mTc-HYNIC-FAPI-04 SPECT/CT in 21 postoperative patients with CRC.

Approximately one quarter of patients who undergo fine-needle aspiration cytology are reported as indeterminate. Ultrasound reporting according to the Thyroid Imaging Reporting and Data System (TI-RADS) helps assess the risk of malignancy in these nodules. Since 2009, when the TIRADS was first proposed, various ultrasound classification systems have been developed by different organizations. Guo et al retrospectively compared the diagnostic value of the American College of Radiology Thyroid Imaging Reporting and Data System ACR-TIRADS, the Korean Society of Thyroid Radiology K-TIRADS, and the Chinese Society of Ultrasound in Medicine C-TIRADS for Bethesda III/IV thyroid nodules in 80 patients.

While the significant majority of the tumours of the liver are hepatocellular carcinoma, about 10% are intrahepatic cholangiocarcinoma. The prognosis of the two differs significantly, and the routine dynamic contrast imaging has limited accuracy in differentiating these tumours. Dai et al conducted a study comparing the diffusion models continuous-time random walk (CTRW), restrictive spectrum imaging (RSI), and diffusion-weighted imaging (DWI), along with their associated histograms, to distinguish subtypes of liver cancer in 40 patients.

Most parotid tumours are benign; however, imaging can aid in differentiating among subtypes. Magnetic resonance imaging is the preferred imaging modality to assess these tumours. However, it may not always be feasible, particularly in patients who are claustrophobic or have metal implants in the body. Gong et al evaluated a nomogram combining quantitative dual-energy computed tomography (DECT) parameters and radiomics to accurately differentiate pleomorphic adenomas (PAs) from Warthin tumours (WTs) of the parotid gland. The authors retrospectively reviewed the imaging data of 120 patients with known histology, dividing them into training and test sets.

It is essential to distinguish pancreatic cancer from benign tumours of the pancreas and mass-forming pancreatitis. The pancreatic lesions are assessed with endoscopic ultrasonography (EUS), which can be supplemented by fine-needle aspiration to establish histology. Fan et al constructed a radiomics and nomogram model based on endoscopic EUS radiomics and clinical data to differentiate benign from malignant lesions. In their study, images from 151 patients were randomly split into training and validation sets.

Mass-forming autoimmune pancreatitis may masquerade as pancreatic cancer due to an overlapping spectrum of symptoms and non-distinctive image findings. Zhang et al developed a differential diagnostic model to distinguish autoimmune pancreatitis (AIP) from pancreatic ductal adenocarcinoma (PDCA) using ultrasound, clinical features and machine learning algorithms. The authors used data from 3 centres as the training cohort, external validation cohort 1, and external validation cohort 2.

Pituitary neuroendocrine tumours are common intracranial tumours, and a small percentage of these tumours may be invasive and infiltrate the surrounding structures. It is crucial to identify the invasion to provide appropriate counselling and surgical planning. Yang et al analyzed data from 133 patients who underwent surgery and had histopathology, and who also underwent multiparameter magnetic resonance imaging (mpMRI) preoperatively. The authors selected 10 radiomics features from 306 primitive features and evaluated the predictive performance of single- and combined-radiomics models for cavernous sinus invasion.

Mariano et al compared the diagnostic performance of Digital Mammography, Breast Ultrasound, Magnetic Resonance Imaging, and Contrast-Enhanced Mammography (CEM) with histopathological confirmation via nipple-areolar complex (NAC) punch biopsies in 2 cases of Paget’s disease. The authors emphasized the diagnostic ability of CEM in this scenario.

Alzheimer’s disease is a neurodegenerative disorder that leads to dementia. It is essential to identify it before the onset of frank symptoms, when the irreversible changes have already occurred. Rauf et al proposed a novel deep learning architecture based on the Inception Module with Self-Attention (InceptionSA) and the Dense Module with Self-Attention (DenseSA). The authors used a depth concatenation layer to fuse these modules at the network level and assessed accuracy on two publicly available datasets.

Perineural spread (PNS) in head and neck malignancy has prognostic and management implications. Efforts have focused on developing a noninvasive tool to identify PNS. One such suggestion was that a three-dimensional (3D) double-echo steady state with water excitation sequence could improve the visualization of the intra-parotid facial nerve. The iso-volumetric 3D imaging sequences can evaluate submillimeter nerves. Extrapolating these, Zheng et al compared three-dimensional liver acquisition with volume acceleration-flexible (3D LAVA_Flex) to two-dimensional magnetic resonance sequences for assessing PNS in nasopharyngeal cancers.

Several biomarkers have been developed to prognosticate nasopharyngeal cancers, including plasma proteins, the neutrophil-to-lymphocyte ratio (NLR), gene-expression markers, and Epstein-Barr virus levels. However, all have some limitations, including the TNM staging methods. In recent years, MRI-based radiomic features have been used to predict patient outcomes. Chen et al evaluated the predictive value of a clinical–radiomics nomogram based on multisequence MRI for advanced NPC outcomes. The authors evaluated MRI scans from 118 patients for whom pre-chemoradiotherapy images were available to calculate a radiomics score, which was used, along with clinical features, to develop the nomogram.

Mixed reality (MR) integrates real-world and virtual elements, thereby advancing augmented reality. It enhances preoperative planning by providing a 3-dimensional visual reconstruction of the tumour and relevant anatomy. Zou et al evaluated the clinical application of mixed reality in robotic nephron-sparing partial nephrectomy for renal tumours. The authors conducted a retrospective analysis of 68 patients who underwent robotic nephron-sparing partial nephrectomy for renal tumours of different complexities. These patients were divided into two groups, with 28 cases in the MR group and 40 cases in the traditional imaging group, and the outcomes were compared.

Bi et al reviewed advances in CT-based lung function imaging for thoracic radiotherapy. The authors analyzed the potential benefits and challenges of CT-based lung function imaging in radiotherapy, including phase-resolved four-dimensional CT (4D-CT) or end-inspiratory and end-expiratory CT scans. Imaging assesses lung function, which can aid in radiotherapy planning and help avoid radiation exposure to healthy lung tissue.

Algorithms have been developed to detect lung nodules in computed tomography scans (18). It is crucial to distinguish malignant from benign lung nodules to ensure appropriate management. Low-dose CT (LDCT) is used for screening in asymptomatic individuals to detect lung nodules. However, it has a limitation in differentiating malignant from benign nodules. Chen et al developed a diagnostic model based on the general condition of the patient, MP outputs from the AI system, the Mayo Clinic model, and serum biomarkers. Data from 260 participants were analyzed, of whom 72 had benign lung nodules, and 188 had early malignant lung nodules. The authors developed a nomogram and conducted a decision curve analysis to construct the model, using 60% of the cohort for training and 40% for validation.

Literature suggests that tumour angiogenesis is an independent prognostic factor for cervical cancer that impacts recurrence, disease-free survival and overall survival. Microvessel density assessment by immunohistochemistry is the gold standard for evaluating tumour angiogenesis. It has limitations, including its invasive nature and tumour heterogeneity, which affect characterization and, in cases treated non-surgically. Zhu et al compared quantified superb microvascular imaging and quantified contrast-enhanced ultrasonography for assessing vascularization in operable versus non-operable uterine cervical cancer.

Overall, this Research Topic is an enriching mix of articles on advancing cancer imaging technologies, which have strong clinical implications and the potential to impact decisions in cancer management. This Research Topic features diverse articles showcasing cutting-edge cancer imaging technologies and research with direct relevance to everyday clinical practice.

## Conclusion

The technological advancement is the future of medicine. It can play a pivotal role in diagnostic imaging and radiomics, treatment planning and assessing treatment response. It can analyze vast patient data and support predictive analytics, which has the potential to change the face of oncology in the era of personalized and precision medicine. AI can also help expand the horizon of drug development by integrating radiomics with genomics to elucidate tumour genetics in the era of targeted therapy, where molecular imaging-based probes can target cell signaling pathways. This can have a profound effect in oncology. However, efforts need to focus on overcoming translational limitations and challenges to harness the full potential of these technological advances.

## References

[1] Chakrabarty N Mahajan A . Imaging analytics using artificial intelligence in oncology: A comprehensive review. Clin Oncol. (2024) 36:498–513. doi: 10.1016/j.clon.2023.09.013, PMID: 37806795

[2] Pallumeera M Giang JC Singh R Pracha NS Makary MS . Evolving and novel applications of artificial intelligence in cancer imaging. Cancers. (2025) 17:1510. doi: 10.3390/cancers17091510, PMID: 40361437 PMC12070983

[3] Mahajan A Burrewar M Agarwal U Kss B Mlv A Guha A . Deep learning based clinico-radiological model for paediatric brain tumor detection and subtype prediction. Explor Target Anti-Tumor Ther. (2023) 4:669–84. doi: 10.37349/etat.2023.00159, PMID: 37720352 PMC10501890

[4] Cheung HMC Rubin D . Challenges and opportunities for artificial intelligence in oncological imaging. Clin Radiol. (2021) 76:728–36. doi: 10.1016/j.crad.2021.03.009, PMID: 33902889 PMC8434981

[5] Bi WL Hosny A Schabath MB Giger ML Birkbak NJ Mehrtash A . Artificial intelligence in cancer imaging: Clinical challenges and applications. CA Cancer J Clin. (2019) 69:127–57. doi: 10.3322/caac.21552, PMID: 30720861 PMC6403009

[6] Mahajan A Vaidya T Gupta A Rane S Gupta S . Artificial intelligence in healthcare in developing nations: The beginning of a transformative journey. Cancer Res Stat Treat. (2019) 2:182. doi: 10.4103/CRST.CRST_50_19

[7] Torigian DA Huang SS Houseni M Alavi A . Functional imaging of cancer with emphasis on molecular techniques. CA Cancer J Clin. (2007) 57:206–24. doi: 10.3322/canjclin.57.4.206, PMID: 17626118

[8] Vaidya T Agrawal A Mahajan S Thakur MH Mahajan A . The continuing evolution of molecular functional imaging in clinical oncology: the road to precision medicine and radiogenomics (Part I). Mol Diagn Ther. (2019) 23:1–26. doi: 10.1007/s40291-018-0366-4, PMID: 30411216

[9] Rutman AM Kuo MD . Radiogenomics: Creating a link between molecular diagnostics and diagnostic imaging. Eur J Radiol. (2009) 70:232–41. doi: 10.1016/j.ejrad.2009.01.050, PMID: 19303233

[10] Bai JW Qiu SQ Zhang GJ . Molecular and functional imaging in cancer-targeted therapy: current applications and future directions. Signal Transduct Target Ther. (2023) 8:89. doi: 10.1038/s41392-023-01366-y, PMID: 36849435 PMC9971190

[11] Mahajan A Goh V Basu S Vaish R Weeks AJ Thakur MH . Bench to bedside molecular functional imaging in translational cancer medicine: to image or to imagine? Clin Radiol. (2015) 70:1060–82. doi: 10.1016/j.crad.2015.06.082, PMID: 26187890

[12] Shur JD Doran SJ Kumar S Ap Dafydd D Downey K O'Connor JPB . Radiomics in oncology: A practical guide. RadioGraphics. (2021) 41:1717–32. doi: 10.1148/rg.2021210037, PMID: 34597235 PMC8501897

[13] Rai P Ahmed S Mahajan A . Radiomics in pediatric brain tumors: from images to insights. Discov Oncol. (2025) 16:1563. doi: 10.1007/s12672-025-03391-5, PMID: 40817158 PMC12356798

[14] Mahajan A Sahu A Ashtekar R Kulkarni T Shukla S Agarwal U . Glioma radiogenomics and artificial intelligence: road to precision cancer medicine. Clin Radiol. (2023) 78:137–49. doi: 10.1016/j.crad.2022.08.138, PMID: 36241568

[15] Mahajan A Kania V Agarwal U Ashtekar R Shukla S Patil VM . Deep-learning-based predictive imaging biomarker model for EGFR mutation status in non-small cell lung cancer from CT imaging. Cancers. (2024) 16:1130. doi: 10.3390/cancers16061130, PMID: 38539465 PMC10968632

[16] Granata V Fusco R Setola SV Santorsola M Ottaiano A Cerrone M . An update of AI and radiomics in precision oncology: insights from liver tumors as case models. Technol Cancer Res Treat. (2025) 24:15330338251387928. doi: 10.1177/15330338251387928, PMID: 41334723 PMC12678745

[17] Xu Y Li Y Wang F Zhang Y Huang D . Addressing the current challenges in the clinical application of AI-based Radiomics for cancer imaging. Front Med. (2025) 12:1674397. doi: 10.3389/fmed.2025.1674397, PMID: 41090135 PMC12515897

[18] Mahajan A Agarwal R Agarwal U Ashtekar RM Komaravolu B Madiraju A . A novel deep learning-based (3D U-net model) automated pulmonary nodule detection tool for CT imaging. Curr Oncol. (2025) 32:95. doi: 10.3390/curroncol32020095, PMID: 39996895 PMC11854842

